# Anti-senescence role of heterozygous fumarate hydratase gene knockout in rat lung fibroblasts *in vitro*

**DOI:** 10.18632/aging.101761

**Published:** 2019-01-21

**Authors:** Zhirui Fan, Lifeng Li, Xiaoli Li, Meng Zhang, Mengmeng Dou, Jing Zhao, Jing Cao, Xiaoming Deng, Mingzhi Zhang, Huixiang Li, Zhenhe Suo

**Affiliations:** ^1^ Department of Oncology, The First Affiliated Hospital of Zhengzhou University, Zhengzhou, Henan Province, China; ^2^ Department of Neurology, The First Affiliated Hospital of Zhengzhou University, Zhengzhou, Henan Province, China; ^3^ Department of Pathology, The Third Affiliated Hospital of Zhengzhou University, Zhengzhou, Henan Province, China; ^4^ Department of Chinese and Western Integrative Medicine, The First Affiliated Hospital of Zhengzhou University, Zhengzhou, China; ^5^ Department of Pathology, The First Affiliated Hospital of Zhengzhou University, Zhengzhou, Henan Province, China; ^6^ Department of Pathology, The Norwegian Radium Hospital, Oslo University Hospital, Institute of Clinical Medicine, University of Oslo, Montebello, Oslo, Norway; ^*^Equal contribution

**Keywords:** anti-senescence, fumarate hydratase, rat lung fibroblasts

## Abstract

Abnormalities in tricarboxylic acid (TCA) cycle function were related to a variety of pathological processes. Fumarate hydratase (FH) is a required enzyme in the TCA cycle. To explore the general influence of FH knockout, we isolated FH^+/–^ rat and normal rat lung fibroblasts and cultured these cells *in vitro*. The isolated fibroblasts with the current method were rather homogeneous and were confirmed spindle in morphology, positive for vimentin and negative for α-SMA (α-smooth muscle actin). Sequencing of the PCR (polymerase chain reaction) products flanking the FH gene mutation verified the FH^+/–^ status, and the FH gene and protein expression were confirmed to be reduced in the FH^+/–^ cells. No sign of ageing for the FH^+/–^ cells after 61 passages was observed, but the controls died out at this stage. Flow cytometry revealed increased S-phase and decreased G1/G0 proportions with significantly less early apoptosis in FH^+/–^ cells compared to that in control cells. At the same time, increased glucose consumption, intracellular fumarate production and extracellular lactate secretion were verified in the FH^+/–^ cells. Correspondingly, FH^+/–^ cells showed a lower basal oxygen consumption rate (OCR) but a higher level of reactive oxygen species (ROS) production. Single cell cloning and cell line establishment were successfully performed with the FH^+/–^ cells at the 84^th^ passage. All the above results indicate an important role for FH^+/–^ in the longevity or immortality of the FH^+/–^ cells, in which increased p53 and TERT (telomerase reverse transcriptase) protein expression, decreased p21 and p16 protein expression and negative SA-β-Gal (senescence-associated beta-galactosidase) were verified along with metabolic reprogramming.

## INTRODUCTION

The TCA cycle is a central hub for energy metabolism, macromolecule synthesis and redox balance. The cycle consists of a series of biochemical reactions occurring in the mitochondrial matrix, which allow aerobic organisms to oxidize fuel sources and provide energy, macromolecules, and redox balance to the cell [1]. Abnormalities in TCA cycle function were related to a variety of pathological processes ranging from cancer to neurological and metabolic disorders. For example, altered expression of citrate synthase (CS), aconitase (ACO2) and malate dehydrogenase (MDH2) has been reported to contribute to cancer-specific features, such as glycolysis addiction, resistance to chemotherapy and increased lipid biosynthesis [2–4]. More importantly, cancer occurrence has been linked to genetic mutations in isocitrate dehydrogenase (IDH) 2, succinate dehydrogenase (SDH) subunits and FH [5].

FH is a required enzyme in the TCA cycle and is responsible for the conversion of fumarate to malate. Mutation in the FH gene leads to the abnormal accumulation of fumarate in cells [6]. This increased fumarate can act as an oncometabolite and promote cancer. Homozygous mutations in the FH gene have previously been reported to cause a severe neurological disorder, and heterozygous germline mutations in the FH gene result in a clinical syndrome characterized by hereditary leiomyomatosis and renal cell cancer [7].

Fibroblasts are the most abundant indigenous cells that are widely distributed in connective tissues and have a variety of functions, including the synthesis of collagen, reorganization of extracellular matrix, and regulation of angiogenesis. Lung fibroblasts differentiate from embryonic mesenchymal cells and constitute the major cellular components of lung connective tissue. Pulmonary fibroblasts have key roles in the formation and maintenance of lung structure and function and are involved in tissue repair and remodelling, providing a certain material basis for lung physiological activity [8].

We established a FH^+/–^ rat model that was reported previously [9]. The purpose of our current study was to explore the impact of heterozygous FH knockout (KO) on metabolic reprogramming, cell biology and ageing in isolated lung fibroblasts in this rat model, with lung fibroblasts from normal rats as controls.

## RESULTS

### Characterization of rat fibroblast cultures

**Figure 1:**
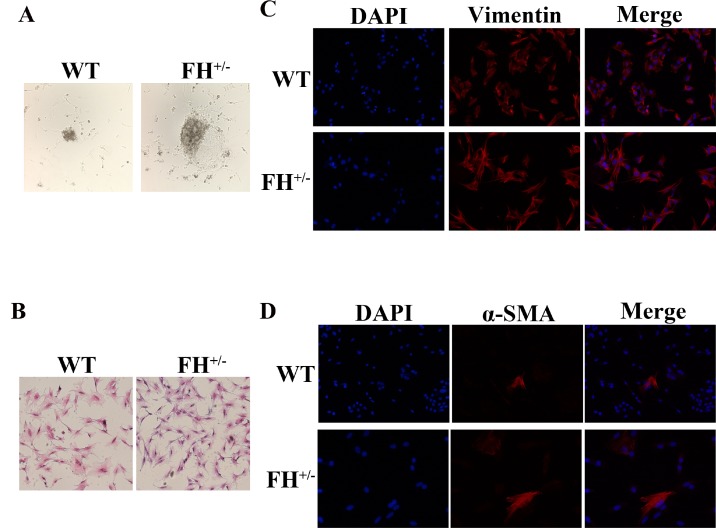
**Characterization of rat fibroblast cultures.** (**A**) The fibroblasts exit from tissue fragments reviewed under a microscope on the 4^th^ day in culture. (**B**) Typical spindle morphology of the fibroblasts evaluated after HE staining. All primary culture cells at the 25^th^ passage were confirmed to be positive for Vimentin by immunofluorescence microscopy (**C**), but occasionally some cells were positive for α-SMA as well (**D**).

The fibroblasts from both the control and heterozygous FH KO rat lungs started to exit tissue fragments within 2–5 days (Figure 1A). All primary rat lung fibroblasts displayed typical spindle-shaped morphology under HE staining (Figure 1B) and were positive for vimentin expression (Figure 1C), but a few cells were positive for α-SMA (Figure 1D), as reported in an previous rat lung fibroblast study [10]. All primary lung fibroblasts grew confluent at approximately five days before passaging. However, after 30 passages, the fibroblasts from the control rats started to slow down the doubling time, from five days before the 30^th^ passage to 12 days after 55 passages. Along with growth slow down, the fibroblasts from the control rats gradually showed a smaller size. These fibroblasts died out at approximately 61 passages (two primary cultures). However, the fibroblasts from the FH^+/–^ KO rats continued the same growth speed without any changes in morphology or doubling time.

### FH^+/–^ KO resulted in reduced FH gene and protein expression

**Figure 2:**
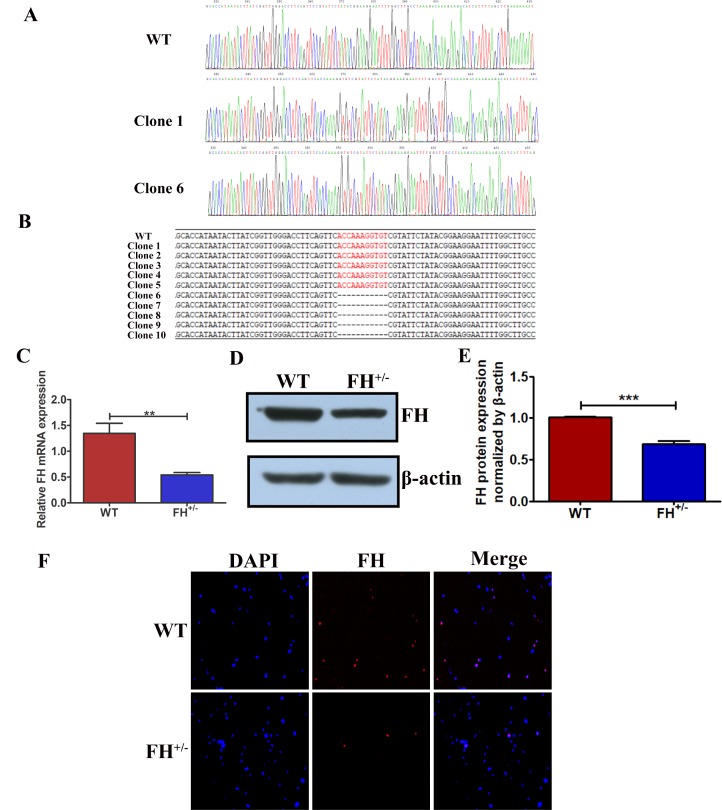
**Reduced FH gene and protein expression in FH^+/–^ cells.** (**A**) FH gene sequencing results for WT and two different situations of FH^+/–^ cells. (**B**) The 11 base pair deletion in one allele in exon 1 in FH^+/–^ cells, which is marked in red in the opposite allele. Significantly reduced FH mRNA expression detected by RT-qPCR is shown in (**C**). Western blot analysis of FH protein expression is shown in (**D**), and the corresponding histogram is shown in (**E**), where the bar graphs represent FH protein expression normalized by β-actin. (**F**) Immunofluorescence microscopy of FH protein expression (200×).

To verify the FH KO in the fibroblasts derived from the FH^+/– ^rats, PCR was performed, and TA cloning of the PCR products were sequenced. The sequences of the TA cloning from both wild type (WT) and FH^+/–^ KO fibroblasts at the 30^th^ passage are shown in Figure 2A. FH^+/–^ cells were identified with 11 base deletions in one allele in FH exon 1. The mutation is shown in Figure 2B and is marked in red. To analyse the FH expression status in the cells, gene and protein expression was detected by real-time quantitative polymerase chain reaction (RT-qPCR) and Western blot and immunofluorescence (IF), respectively. The FH mRNA expression was reduced by approximately 59.66% in the FH^+/– ^cells compared to that in the controls, as verified by the RT-qPCR results shown in Figure 2C. Western blot analysis of the cells demonstrated approximately 31.90% reduced FH protein expression in the FH^+/–^ cells, as shown in Figure 2D and 2E. Figure 2F also shows decreased protein expression in the FH^+/–^ cells.

### FH^+/–^ KO accelerated cell proliferation

**Figure 3:**
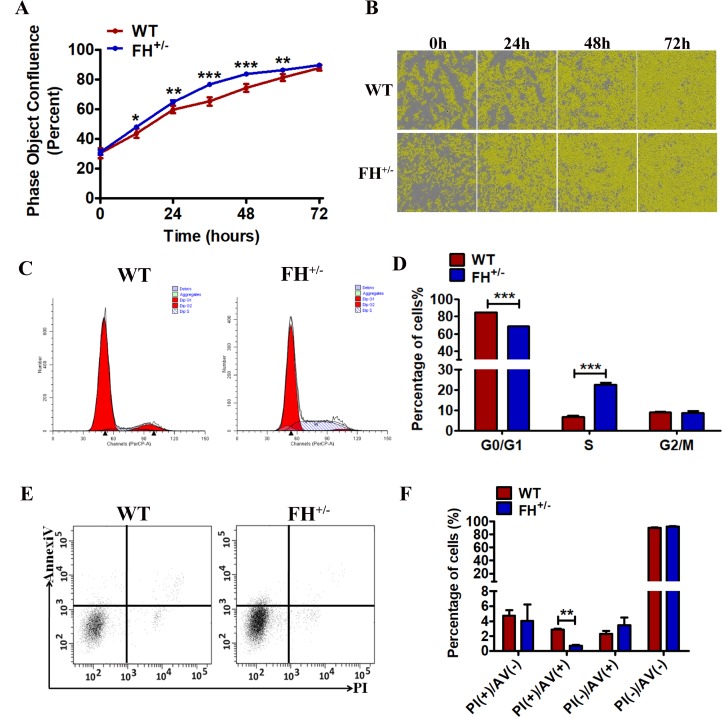
**Effect of FH^+/–^ KO on proliferation, apoptosis and cell cycle.** All experiments were performed on the cells at approximately 25 passages. (**A**) and (**B**) The growth curves and images, respectively, generated by the Incucyte Zoom system. A representative flow cytometry cell cycle analysis is shown in (**C**), and the corresponding histogram of the cell cycle analysis is shown in (**D**), where the bar graphs represent the percentage of WT and FH^+/–^ cells in the G2/M, S and G0/G1 phases. Early apoptosis detected by flow cytometry is shown in (**E**), and the corresponding histogram is shown in (**F**), where the bar graphs represent the percentage of apoptotic cells.

To determine whether the FH KO had an effect on cell proliferation, we explored cell growth analyses. Growth curves demonstrated that the growth of FH^+/–^ cells was always faster compared with that of WT cells at the same passage (Figure 3A and 3B). For further analysis, cell cycle and cell apoptosis were detected by flow cytometry. As shown in Figure 3C and 3D, the FH^+/– ^cells show a significantly increased percentage of cells at S phase (p< 0.001) and a decreased percentage of cells at the G0/G1 phases (p< 0.001) compared with the WT cells, indicating G0/G1 phase arrest in the WT cells. The results of the apoptosis assay are shown in Figure 3E and 3F. The early apoptosis of WT cells was significantly higher than that of FH^+/–^ cells.

### FH^+/–^ KO impaired the oxidative TCA cycle and promoted anaerobic glycolysis

**Figure 4:**
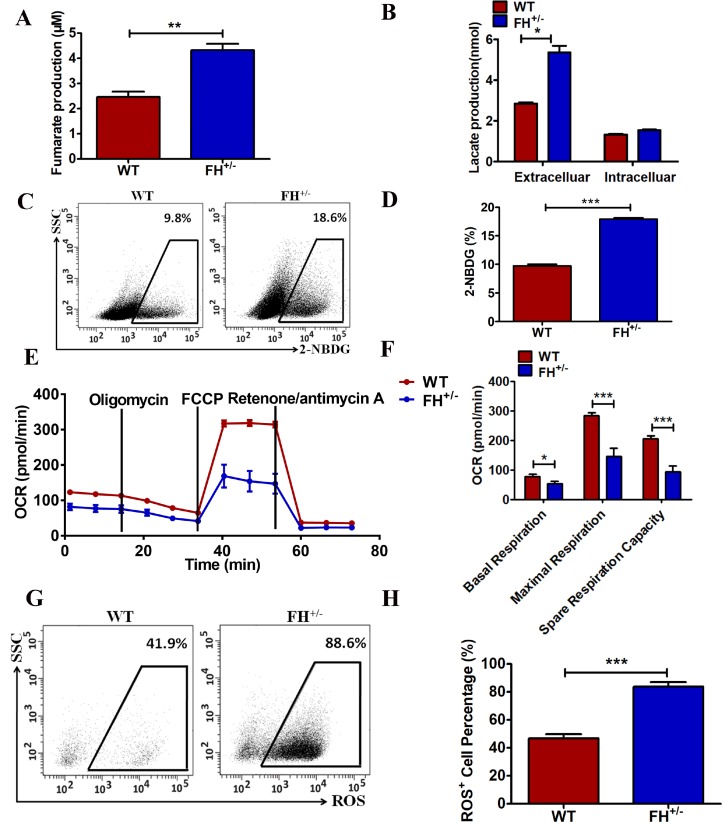
**Metabolic reprogramming of the FH^+/–^ cells.** (**A**) Significantly more cellular fumarate production, and the bar graphs represent the fumarate concentration detected with the assay kit. (**B**) Extracellular lactate excretion and cytoplasmic lactate production. (**C**) Representative glucose consumption results examined by flow cytometry, and the corresponding histogram is shown in (**D**). (**E**) Representative OCR results investigated by a Seahorse XFe96 Extracellular Flux Analyser and (**F**) the OCR histograms. A representative flow cytometry of ROS is shown in (**G**), and the corresponding histogram is shown in (**H**), where the bar graphs show the percentages of ROS^+^ cells among the WT and FH^+/–^ cells.

To further confirm the functional alteration of the FH^+/–^ cells, the fumarate concentration in these cells was detected. As shown in Figure 4A, FH^+/–^ cells show a significantly larger amount of cellular fumarate (p=0.05) compared to the WT cells, indicating the impairment of the catalytic function of fumarase in these cells. We next asked whether there were metabolic changes in the FH^+/–^ cells. First, the difference in lactate acid secretion and cellular retention in the FH^+/–^ cells was examined. There was a significantly larger amount of extracellular lactate acid in the FH^+/– ^cells compared to that in the WT cells, as shown in Figure 4B (p=0.01). However, there was no significant difference in the cellular lactate acid concentration in the FH^+/–^ cells compared with that in WT cells, as shown in Figure 4B (p=0.09), although higher levels of cellular lactate in the FH^+/–^ cells could be detected. We then examined glucose consumption in these cells. The FH^+/–^ cells consumed significantly more glucose (p=0.000) compared with the control cells, as shown in Figure 4C and 4D.

To further reveal the status of metabolism in the FH^+/–^ cells, functional mitochondria analyses were performed by using a Seahorse XFe96 Extracellular Flux Analyser, focusing on the OCR under basal and stressed conditions in the presence of oligomycin, phenylhydrazone (FCCP) and rotenone/antimycin A. Under basal conditions, the OCR of FH^+/–^ cells was 81.8±9.1 pmol/min per 5×10^4^ cells, which was significantly lower than that in the WT cells (123.3±4.3 pmol/min, p=0.03) (Figure 4E and 4F). The addition of FCCP should lead to an increase in OCR in cells. The experiments showed that a concomitant OCR increase (283.28±3.7 pmol/min) was observed in the WT cells, while under the same stress conditions, only a slight increase in OCR (146.33±4.7 pmol/min) was observed in the FH^+/–^ cells (Figure 4E and 4F, p=0.000). The FCCP-induced OCR increase represents respiratory reserve capability, as shown in Figure 4F, which shows significantly reduced spare respiratory capability in the FH^+/–^ cells (p=0.000), implying that at basal level, the FH^+/–^ cells operated at approximately maximal OCR. Therefore, the FH^+/–^ cells showed defective mitochondrial function, indicated by low basal OCR and a lack of response to FCCP. To reveal the effect of FH^+/–^ on ROS generation, ROS detection was performed. There was significantly higher ROS in the FH^+/–^ cells compared to that in the WT cells, as shown in Figure 4G and 4H (P=0.001). In summary, these experiments demonstrated abnormal mitochondrial oxidative respiration in the FH^+/–^ cells.

### Transcriptomic analysis

**Figure 5:**
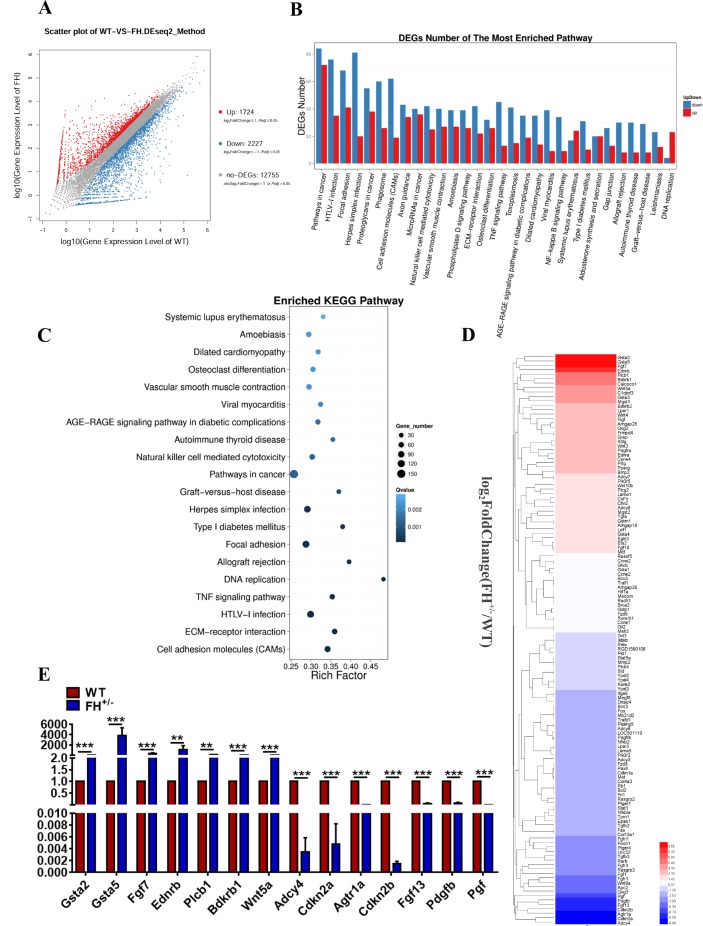
**Results of transcriptome sequencing.** (**A**) Scatter plots of all expressed genes by BGISEQ-500. Blue represents downregulated genes, red represents upregulated genes and brown represents non-regulated genes. (**B**) and (**C**) The results of the pathway function analysis of differentially expressed genes. The up and down of differentially expressed genes is shown in (**B**), where the X-axis indicates pathway entry, the Y-axis represents the number of genes and up and down for the corresponding pathway entry. (**C**) The results of differential gene pathway enrichment, where the X-axis represents the enrichment factor and the Y-axis represents the pathway name. (**D**) Genes of the pathways in cancer. The RT-qPCR validation of the selected DEGs revealed by transcriptome sequencing is shown in (**E**).

To explore the transcriptomic difference between the FH^+/–^ and control fibroblasts, 6 samples, including 3 WT and 3 FH^+/–^ at the 50^th^ passage, were subjected to transcriptome sequencing technology. The scatter plots of all expressed genes based on the results of each WT-FH^+/–^ pair are shown in Figure 5A. A total of 1724 significantly upregulated genes and 2227 downregulated genes compared with WT were screened under the criteria of a minimum 2-fold change and a P value < 0.05.

Genes usually interact with each other to play roles in certain biological functions. Therefore, we performed a pathway enrichment analysis of differentially expressed genes (DEGs) based on the Kyoto Encyclopaedia of Genes and Genomes (KEGG) database. Pathways in cancer had the largest number of DEGs (Figure 5B and 5C). The genes in cancer pathways are shown in Figure 5D. To verify the results of transcriptome sequencing analyses, 14 DEGs associated with pathways in cancer based on the above transcriptome sequencing analyses were selected for RT-qPCR to further investigate their expression profiles. Gene expression measured by RT-qPCR exhibited similar changes to the gene expression profiles, with 7 genes (Gsta2, Gsta5, Fgf7, Ednrb, Plcb1, Bdkrb1, and Wnt5a) upregulated and 7 genes (Adcy4, Cdkn2a, Agtr1a, Cdkn2b, Fgf13, Pdgfb, and Pgf) downregulated (Figure 5E). Among these genes, Gsta2 showed the largest upregulation, and Adcy4 manifested the largest downregulation. Other genes of interest from the sequencing analysis included Cdkn2a and Cdkn2b, which are tumour suppressor genes that showed large downregulation by RT-qPCR.

### Immortality of FH^+/–^ cells

**Figure 6:**
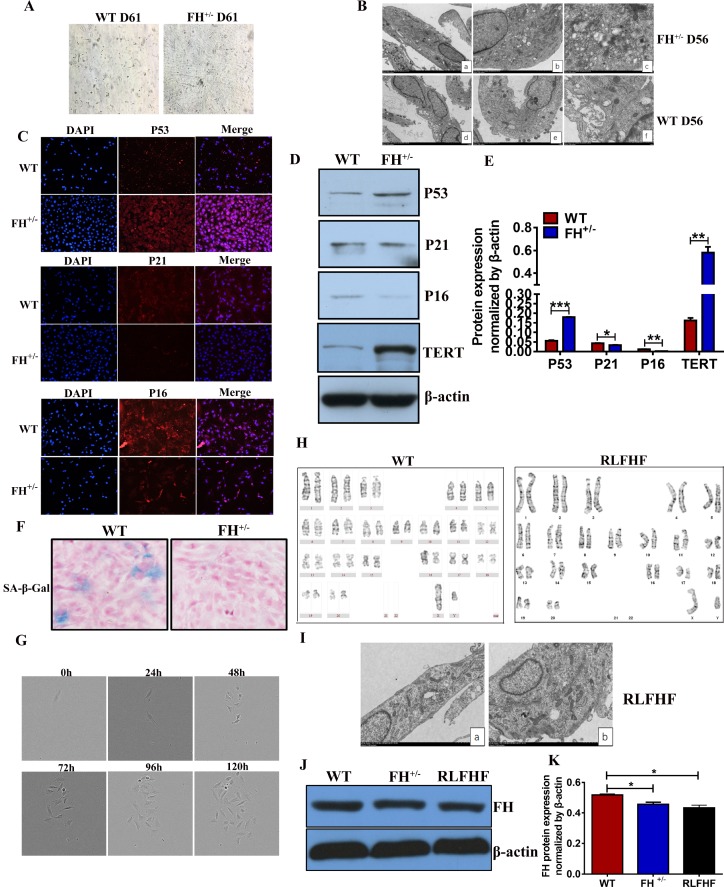
**Further characterization of the FH^+/–^ cells.** (**A**) Microscopic images of WT and FH^+/–^ cells at the 61^st^ passage (200×). (**B**) Representative ultrastructure images of WT and FH^+/–^ cells at the 55^th^ passage evaluated by electron microscopy (a,d:2000×; b,e:4000×; c.f:8000×), where a larger cell body with plenty of rough endoplasmic reticulum, mitochondria and ribosomes is shown in the FH^+/–^ cells, but the WT fibroblasts show fewer mitochondria and ribosomes and deformed and less condensed rough endoplasmic reticulum. (**C**) Representative immunofluorescence images of the expression of p53, p21 and p16 (200×). Western blot for p53, p21, p16 and TERT in WT and FH^+/–^ cells and the corresponding histograms are shown in (**D**) and (**E**), respectively. (**F**) SA-β-Gal detection . (**G**) FH^+/– ^single cell cloning images captured with the Incucyte Zoom System for cells at the 84^th^ passage. (**H**) The karyotype of a RLFHF FH^+/–^ cell, where no chromosome abnormality is seen as revealed for the karyotype of a control fibroblast at the 40^th^ passage. (**I**) Electron microscope images of RLFHF FH^+/–^ cells (4000× for both a and b), which are rich in mitochondria, rough endoplasmic reticulum and ribosomes. Western blot for FH protein expression in the 58^th^ passage WT, 68^th^ passage FH^+/–^ and RLFHF cells is shown in (**J**) and corresponding histograms are shown in (**K**).

We noted that when the FH^+/–^ cells were constantly grown under the same culture conditions from the first generation to approximately the 60^th^ generation without change, the fibroblasts from the control rats not only became gradually slower in growth but also showed smaller bodies in size, indicating a typical apoptotic process (Figure 6A).

To study the ultrastructural changes of the cells, we performed transmission electron microscopy on the WT and FH^+/–^ cells at passage 55. As shown in Figure 6B, the FH^+/–^ cells are usually larger with prominent cellular organelles, such as mitochondria, Golgi complex, rough endoplasmic reticulum and ribosomes. In contrast to these cells, the control fibroblasts show smaller body sizes and fewer prominent organelles, such as mitochondria, Golgi complexes, rough endoplasmic reticulum and ribosomes. Occasionally, a large rough endoplasmic reticulum could be observed, but the reticulum cavity was extremely enlarged, indicating a problem with secretion or material transportation. To observe whether the WT cells show senescence, we examined the protein expression of p53, p21, p16 and the activity of SA-β-Gal in both groups at passage 50. The results showed that p53 and TERT protein expression in the FH^+/–^ cells was upregulated, whereas p21 and p16 were downregulated (Figure 6C, 6D and 6E). As shown in Figure 6F, WT cells were positive for SA-β-Gal, but no SA-β-Gal positive cells were seen in the FH^+/–^ cells.

All fibroblasts derived from the control rats died out at passage 61, while the fibroblasts derived from the FH^+/–^ rats demonstrated no signs of ageing. After 84 passages for the FH^+/–^ cells, we performed single cell cloning with these fibroblasts. Single cells from the FH^+/–^ fibroblasts could continuously divide and finally form a clone in the 96-well plate, as shown in Figure 6G, which could be expanded in larger plates and conventional cell culture flasks later. Three clones were successfully obtained, and one clone was continuously propagated at the 25^th^ passage, and the line was then named RLFHF. Two other clones were frozen at the 5^th^ passage. Further karyotyping and electron microscopic analyses of the RLFHF cells were performed. As shown in Figure 6H, there are 21 pairs of chromosomes in the FH^+/–^ cells with no abnormal chromosomal morphological structure. Electron microscopy of the FH^+/–^ cells revealed a similar ultrastructure as shown at an earlier 55^th^ passage, with prominent cell organelles and a high density of ribosomes (Figure 6I). To understand the FH expression in the later passages and RLFHF clone, Western blot was performed, and FH expression in the FH^+/– ^and RLFHF cells was significantly lower than that in the 58^th^ passage WT cells (Figure 6J and 6K).

## DISCUSSION

In recent years, the relationship between mitochondrial function and cell survival, especially cancer cell immortality, has attracted widespread attention [11–13]. Cancer cells, in contrast to normally differentiated cells, which primarily utilize mitochondrial oxidative phosphorylation to generate the energy for cellular bioactivities, rely on largely aerobic glycolysis, a phenomenon termed “the Warburg effect” [3]. Mitochondria are conventionally viewed as the cell ‘energy factory’, as these organelles normally generate almost 90% of cellular ATP through the process of oxidative phosphorylation and provide the energy needed for the metabolism of various substances in cells [14]. The metabolic functions of the mitochondria include the TCA cycle, oxidative phosphorylation (OXPHOS), generation of biosynthetic precursors, catabolism of fatty acids and amino acids, and redox regulation [15]. Research has shown that normal mitochondrial function can suppress tumourigenesis [16]. The Warburg effect describes a form of mitochondrial dysfunction such that cancer cells are able to bypass mitochondrial oxidative phosphorylation under stress conditions [3].

The TCA cycle is a key metabolic pathway that allows mammalian cells to use glucose, amino acids, and fatty acids. The entry of these fuels into the cycle is carefully regulated to efficiently fulfil the bioenergetics and biosynthetic, and redox balance requirements of cells. Multiple types of cancers are marked by drastic changes in TCA cycle enzymes, which result in characteristic metabolic and epigenetic changes that are correlated with disease transformation and progression [7, 17–19]. Inactivating mutations in FH result in fumarate accumulation and metabolic reprograming [20], which includes an increased dependence on glycolysis and glutamine anaplerosis [21].

In our study, we successfully cultivated FH^+/–^ lung fibroblasts with the fibroblasts from WT rats as controls and discovered no sign of ageing for the FH^+/–^ lung fibroblasts after 61 passages, but all control fibroblasts died out at this stage. The isolated fibroblasts with the current method were rather homogeneous and were confirmed spindle in morphology, positive for vimentin and negative for α-SMA, supportive of fibroblasts. PCR products flanking the FH^+/–^ mutation were sequenced three times with identical mutations as reported earlier [9]. Furthermore, diminished FH gene and protein expression was observed in the FH^+/–^ cells. Flow cytometry revealed increased S-phase and decreased G1/G0 proportions with early apoptosis compared to that in the control cells. Additionally, increased intracellular fumarate production, glucose consumption and extracellular lactate secretion were verified in the FH^+/–^ cells. Correspondingly, the FH^+/–^ cells showed less basal OCR but higher levels of ROS production. The above results indicate an important role for FH^+/–^ in the longevity or immortality of FH^+/–^ cells, which is interesting for both ageing and cancer studies.

FH is known as a TCA cycle enzyme and tumour suppressor, and the deletion of FH leads to cellular fumarate accumulation and TCA cycle blockage [22, 23]. The energy metabolism of cells is achieved by both the cellular glycolytic pathway and the oxidative phosphorylation of carboxylic acid in mitochondria. There is mutual cooperation and competition between these two pathways. Generally, if the oxidative phosphorylation pathway of the carboxylic acid in mitochondria is weakened, then the cells will upregulate the glycolytic pathway through some mechanisms [24]. Studies have shown that defects in mitochondrial function can cause the reprogramming of energy metabolism and promote the occurrence and growth of tumours through some mechanisms [25–28]. The results of ROS and OCR assays demonstrate that the mitochondrial function of the FH^+/–^ cells is defective. Increased extracellular lactic acid and enhanced cellular glucose uptake indicate an increased cell glycolysis rate. These phenomena indicate that FH^+/– ^cells have mitochondrial dysfunction and are forced to rely on glycolysis to produce energy. Correspondingly, transcriptome analysis revealed a series of gene alterations in the cancer pathways.

As a tumour suppressor, p53 is one of the most commonly inactivated or mutated genes in human cancer [29]. Loss of p53 function through mutations is a common feature in the majority of human cancers, and more than 75% of the mutations result in the expression of a p53 protein that has, in most cases, lost wild-type functions [30]. Recently, it was also reported that dimethyl fumarate cytoprotection against oxidative stress in astrocyte culture *in vitro* is due to p53 expression induction via NRF2-dependent OSGIN1 expression [31], indicating an important role for cytoplasmic fumarate accumulation. Notably, p53 plays a central role in not only cancer suppression and stem cell protection but also in anti-ageing [32]. However, there may be mechanisms other than NRF2 and OSGIN1 underlying the p53 induction in FH^+/–^ fibroblasts because we could not identify any expression variation of these genes in our current study. As a cyclin-dependent kinase (CDK) inhibitor, p21 plays an important role in cell cycle arrest and senescence [33–35]. Alterations of p21 are also associated with ageing, cellular senescence, cellular differentiation and tumourigenesis [35–37]. Another tumour suppressor gene, p16, encodes a specific inhibitor of CDK4 and CDK6 and is altered in a wide range of human cancers [38]. Cellular senescence is a stable cell cycle arrest caused by damage, such as oncogene activation, irradiation, DNA damage, oxidative stress, viral infection and the abrogation of tumour suppressor gene functions [35, 39, 40]. Cellular senescence is a barrier to oncogenic transformation induced by oncogenic signals, the abrogation of which enables the path to tumourigenesis [41, 42]. Growing evidence suggests that senescence and tumourigenesis are crossed during tumour progression [43]. During the past 20 years, the SA-β-gal assay has been viewed as the gold standard for identification of senescent cells [44–46] and has been the most extensively utilized biomarker for senescent cells in vitro and in vivo [47, 48]. It was reported that TERT was able to improve mitochondrial function and to decrease oxidative stress which was associated with cellular senescence and ageing [49, 50]. Compared to the control cells, the p21, p16 protein expression in the FH^+/– ^cells was significantly downregulated, but P53 and TERT were upregulated, together with the negative SA-β-Gal strongly indicating an important role for FH^+/– ^induced anti-ageing cascades, which include at least metabolic reprogramming and fumarate-induced signalling.

In summary, we have confirmed the longevity/immortality of the fibroblasts derived from the FH^+/–^ Sprague-Dawley (SD) rat lungs, while the fibroblasts from the control SD rat lungs can survive only up to 61 passages in the current study. The FH^+/–^ cells grow faster and show significantly increased S-phase and decreased G1/G0 proportions with significantly less early apoptosis. Furthermore, the FH^+/–^ cells demonstrated prominent metabolic reprogramming towards the Warburg effect and significantly increased cellular fumarate and gene alterations in cancer pathways, including the increased expression of p53 and TERT, the decreased expression of p21, p16 and negative SA-β-Gal. All of these results support a potential role for FH^+/– ^in anti-ageing and therefore carcinogenesis as well.

## MATERIALS AND METHODS

### Fibroblast isolation and culture

SD rat lungs were removed and placed in 50 ml tubes with sterile phosphate buffer saline (PBS) to avoid drying after the rats were euthanized with 10% chloral hydrate (3 males and 3 females at 2 months). The lungs were then transferred into a 10 cm tissue culture dish, cut into 1 mm^3^ pieces using two sterile scalpels and 10 ml of DMEM/F12 media with 2 mg/ml collagenase (C5138, Sigma, USA), and 1X antibiotic/antimycotic was added and the sample was stirred slowly at 37°C for 90 mins. The solution containing tissue fragments in the dish was pipetted up and down to breakdown the tissue clumps before transferring to a 50 ml sterile tube, followed by centrifuging for 5 min at 1000 rpm to remove the supernatant. The tissues were washed 3 times with 30 ml DMEM/F12 media supplemented with 15% FBS and 1X antibiotic/antimycotic to remove the traces of collagenase. The pellet was resuspended in 2 ml of MEM/EBSS supplemented with 15% FBS and 1X antibiotic/antimycotic and transferred to culture bottles before being placed in a culture incubator at 37°C and 5% CO_2_. The bottles were checked and the media was changed every day.

### Haematoxylin and eosin (HE) staining

A total of 2×10^4^ cells were grown on coverslips, which were placed on the bottom of 12-well plates. The cells at 75% confluence were fixed with 4% paraformaldehyde in PBS (pH 7.5) for 30 min, stained with haematoxylin for 3 min, rinsed under running tap water, stained with eosin for 3 mins, dehydrated with anhydrous ethanol, transparentized with xylene I and xylene II, and finally mounted in neutral gum. The cells were observed under a microscope.

### Immunofluorescence microscopy

The cells were grown on coverslips in 6-well plates until 75% confluence. Before examination, the cells were fixed with 4% paraformaldehyde in PBS (pH 7.5) for 30 min and permeabilized with 0.5% Trion X-100 for 15 min at room temperature. The coverslips were first immersed for 1 h in blocking solution containing 5% bovine serum albumin (BSA) in PBS, and then the cells were incubated overnight at 4°C with antibodies against Vimentin (ProteinTech Group, China), α-SMA (ProteinTech Group, China) FH (ABCAM, UK), p53 (Abcam, UK), p21 (Abcam, UK) and p16 (ABCAM, UK). The DNA was counterstained with 4’, 6-diamidino-2-phenylindole (DAPI, 5 µg/ml) and observed under an inverted fluorescence microscope.

### Mutation analysis (Genomic DNA extraction, PCR, restriction digests assay and sequencing)

A total of 5×10^6^ cells at 75% confluence were harvested for genomic DNA isolation with the Tissue DNA Kit (OMEGA, D3396-02, USA) following the manufacturer’s instructions. The extracted DNA was amplified by PCR in a total volume of 25 µl containing 2X Taq Master Mix (CW Biotech, Beijing, China) 12.5 µl, forward primer 1 µl, reverse primer 1 µl, DNA 500 ng and RNase free water. The primers used are shown in Table 1. The conditions for PCR were as follows: 98°C for 3 mins; 98°C for 10 s, 60°C for 20 s and 72°C for 20 s for 35 cycles; 72°C for 10 mins; and a 4°C hold. The PCR products were cloned into the pMD19-T vector (D102A, TaKaRa, China) and sent to BGI (Shenzhen, Guangdong, China) for sequencing.

### RT-qPCR analysis

Total RNA from 1×10^6^ cells was extracted using TRIZOL reagent (TaKaRa, Japan). The concentration and purity of all total RNA samples was verified using the NanoDrop 2000 (Thermo Scientific). The Revert Aid First Strand cDNA Synthesis Kit (TaKaRa, Japan) was used for cDNA synthesis. The forward and reverse primer sequences are shown in Table 2. Quantitative real-time PCR using a SYBR Green PCR master mix (Qiagen, Germany) was performed with the Agilent Mx3005P. The samples were amplified using the following conditions: 95°C for 5 min and 40 cycles of 95°C for 10 sec and 60°C for 30 sec. Relative gene expression was determined by normalizing the expression of each target gene to GAPDH. The data were analysed by using 2^-ΔΔCt^.

**Table 1:** Primers for sequencing

Gene Name		Sequence(5ʹ to 3ʹ)
FH	Forward primer	CTGGGCAGTATGTGAATTGTATAAAC
	Reverse primer	GAACCCTGACTAAAACAGCCC

### Western blot

A total of 1×10^6^ cells at 75% confluence were harvested and lysed with RIPA buffer containing 50 mM Tris-HCl (pH 7.5), 150 mM NaCl, 1% NP-40, 0.5% sodium deoxycholate, 0.1% SDS and 1% PMSF (100×)(CW Biotech, Beijing, China) on ice for approximately 30 mins and then centrifuged at 1500 g for five mins at 4°C. Protein samples (30 μg) were loaded onto 12% SDS-PAGE gels and then transferred to PVDF (Millipore, Bedford, MA) membranes. The membranes were blocked in TBST buffer containing 5% non-fat milk at room temperature (22°C) for 1 h and then probed with antibodies against FH (11375-1-AP, ProteinTech Group, China), p53 (ab131442, Abcam, UK), p21 (ab109199, Abcam, UK), p16 (ab51243, ABCAM, UK) and TERT(bs-0233R, Bioss, China ) at 4°C overnight, followed by incubation with the respective horseradish peroxidase-conjugated secondary antibodies for 1 h at room temperature. The membranes were exposed to a chemiluminescent reagent (ECL) for approximately 5−10 min. The membranes were then exposed to X-ray photographic film in a darkroom, and the band densities were later quantified with ImageJ software (NIH, USA).

### Cell proliferation assay

Real-time assessment of cell proliferation over 3 days was carried out using the IncuCyte Imaging System (Essen Biosciences). The cells were seeded onto 96-well plates (35599, Corning, USA) with 3000 cells per well and incubated in medium containing 10% FBS overnight before being transferred to the IncuCyte apparatus. Images (2/well) were collected every 4 h over time, and cell growth curves were obtained with the corresponding software installed in the IncuCyte System.

### Cell cycle analysis

After harvesting the cells with 0.05% trypsin without EDTA solution, single cell suspensions were fixed using ice-cold 75% ethanol at 4°C overnight. For cell cycle analysis, the cells were stained with 2 μg/ml RNase A and 10 μg/ml PI in 500 μl PBS. The DNA contents were measured with the BD LSR II flow cytometer, and FlowJo version 7.6 software was used for further data analysis.

### Flow cytometric evaluation of apoptosis

A total of 10^6^ cells at 75% confluence were harvested and washed twice with ice-cold PBS. Then, the cells were resuspended in Annexin V-binding buffer. Thereafter, the cells were incubated with Alexa Fluor 647 Annexin V (cat no. 640912, Biolegend, USA) for 15 min at 4°C in the dark, and PI (Sigma, USA) was subsequently added. The samples were immediately analysed by flow cytometry (BD FACSCanto II).

### Fumarate measurement

The concentrations of cellular fumarate were determined according to the manufacturer’s instructions (Fumarate Colorimetric Assay Kit, Biovision, K633-100, USA). Briefly, 1×10^6^ cells at confluence were homogenized in the assay buffer and centrifuged at 13,000 g for 10 min to remove insoluble materials. A total of 30 µl of the sample supernatant with 70 µl of reaction buffer was added to a 96-well plate. After a 1-h incubation at 37°C, the absorbance of the samples and the standard were measured at an optical density (OD) of 450 nm.

### Lactate assay

A total of 2×10^5^ cells were seeded evenly onto 6-well plates with 3 ml of medium and cultured for 48 h. Then, the cell culture medium was collected and centrifuged at 13,000 g for 10 mins. The supernatant was then use in the extracellular lactate assay with the Lactate Assay Kit (MAK064, Sigma, USA). Additionally, the cells were harvested and homogenized in assay buffer and centrifuged at 13,000 g for 10 min to remove insoluble materials, and the supernatant was used in the intracellular lactate assay with the same kit. To perform the lactate assay, 50 µl of sample supernatant with 50 µl of master reaction mix was added to a 96-well plate. OD values at 570 nm were read after 30 mins of incubation at room temperature. The sample lactate acid concentration was determined by the OD values from the standard curve.

**Table 2:** Primers for RT-qPCR

Gene Name		Sequence(5ʹ to 3ʹ)
FH	Forward primer	TGTGAGGCTGTTGATTGGA
	Reverse primer	GGCGAAACAAACCACAGTTC
Gsta2	Forward primer	AGTGCATCCGGTGGCTCCTG
	Reverse primer	GGTGGCGATGTAGTTGAGAATGGC
Gsta5	Forward primer	CTACCTTGTAGGCAACAGGCTGAC
	Reverse primer	AGGCTGCTGATTCTGCTCTTGAAG
Fgf7	Forward primer	ATCCTGCCGACTCCGCTCTAC
	Reverse primer	TCTGCTCTGGACTCATGTCATTGC
Ednrb	Forward primer	CCAACTCCAGTCTGATGCGTTCC
	Reverse primer	AGCACGAACACGAGGCATGATAC
Plcb1	Forward primer	CCTCAGCTCTTCTGGAATGC
	Reverse primer	CAGCCTGTAGCCACTCTTCC
Bdkrb1	Forward primer	TGGCTATCAGTCAGGACCGCTAC
	Reverse primer	GATGCAGGCAGAGACGTTCAGATC
Wnt5a	Forward primer	CAAATAGGCAGCCGAGAGAC
	Reverse primer	TGCAACCACAGGTAGACAGC
Adcy4	Forward primer	GCTGTCCGACTGCCTCATTGC
	Reverse primer	AGCCAGGACGAGGAGTGTGAAG
Cdkn2a	Forward primer	CAGCTCTCCTGCTCTCCTATGGTG
	Reverse primer	CAGGCATCGCGCACATCCAG
Agtr1a	Forward primer	GCTTCAACCTCTACGCCAGTGTG
	Reverse primer	CAGCCAGATGATGATGCAGGTGAC
Cdkn2b	Forward primer	CCAACGCCGTCAACCGCTTC
	Reverse primer	TGCCTTGTGCAGCACCATTAGC
Fgf13	Forward primer	GGAAGTCGTATTCAGAGCCTCAGC
	Reverse primer	TGGTGCCATCAATGGTTCCATCTG
Pdgfb	Forward primer	TCTCTGCTGCTACCTGCGTCTG
	Reverse primer	AAGGAGCGGATGGAGTGGTCAC
Pgf	Forward primer	TGTCCTTCTGAGTCGCTGTAGTGG
	Reverse primer	CATTCGCAGAGCACATCCTGAGAG

### Glucose uptake assay

Glucose uptake was measured by flow cytometry according to the manufacturer’s instructions (2-NBDG, Cayman, 186689-07-6). The cells were collected, washed in PBS, and then resuspended in glucose-free medium containing 100 μM 2-deoxy-2- [(7-nitro-2, 1,3-benzoxadiazol-4-yl) amino]-D-glucose (2-NBDG). After incubation for 2 h, the cells were collected, and the fluorescence was measured by flow cytometry.

### OCR analyses

OCR was measured using a Seahorse XF^e^96 Extracellular Flux Analyser (Seahorse Bioscience, North Billerica, MA, USA). Briefly, the cells were plated at 5×10^4^ cells/well onto an XF^e^96 cell culture microplate (Seahorse Bioscience) and allowed to attach to the bottom overnight. The cell culture medium was changed to analysis medium supplemented with 4 mM glucose, 2 mM sodium pyruvate and 2 mM glutamine at 1 h prior to the beginning of the assay and maintained at 37°C. After baseline measurements, OCR was measured by sequentially injecting each well with oligomycin (1 µM), carbonyl cyanide-4 (trifluoromethoxy), phenylhydrazone (FCCP)(1 µM) and rotenone/antimycin A (0.5 µM). The values were calculated as absolute OCR in pmol O_2_/min. Each measurement loop comprised 30 sec mixing, 3 min waiting and 3 min measuring of oxygen consumption. The OCR data were corrected for cell number by nuclear staining with Hoechst.

### ROS assay

Intracellular ROS production was detected using the fluorescent probe DCFH-DA. The cells were pretreated with 4 μmol/L carboxy-2’, 7’-dichlorodihydrofluorescein diacetate (H2DCFDA; Sigma) and then stained with surface markers to monitor the ROS level in the cells.

### Transcriptomic analysis

Six samples (three WT cells and three FH^+/–^ cells) were sequenced at BGI (Shenzhen, Guangdong, China) using transcriptome sequencing technology. The first step in the workflow involved purifying the poly-A-containing mRNA molecules using poly-T oligo-attached magnetic beads. Following purification, the mRNA was fragmented into small pieces using divalent cations under elevated temperature. The cleaved RNA fragments were copied into first strand cDNA using reverse transcriptase and random primers. This was followed by second strand cDNA synthesis using DNA polymerase I and RNase H. These cDNA fragments underwent the addition of a single ‘A’ base and subsequent ligation of the adapter. The products were then purified and enriched through PCR amplification. We then quantified the PCR yield by Qubit and pooled the samples together to make a single strand DNA circle (ssDNA circle), which generated the final library. DNA nanoballs (DNBs) were generated with the ssDNA circle by rolling circle replication (RCR) to enlarge the fluorescent signals during the sequencing process. The DNBs were loaded into the patterned nanoarrays, and pair-end reads of 100 bp were read on the BGISEQ-500 platform for the following data analysis.

### SA-β-Gal staining

The cells were grown on coverslips in 6-well plates until 75% confluence. Then the staining was performed according to the manufacturer’s instructions (G1073-1, Sigma, USA). The cells were observed under a microscope and positive cells were stained blue.

### Single cell cloning

The cell suspension was prepared, and the cell number was counted before the sample was serially diluted to a final concentration of 7 cells per ml. The suspension was dispensed into 96-well plates at 100 µL per well (average 0.7 cells per well) and incubated at 37°C and 5% CO_2_. After overnight cultivation, the cells were observed under a microscope, and the wells were confirmed to contain only a single labelled cell. The next day, 100 µl of culture medium was added to the labelled wells. When the cells reached density saturation (clone), the wells still containing single clones were digested with trypsin, and the detached cells from the single clones were transferred to 24-well plates. When the cells in the 24-well plates reached confluence, they were transferred to 6-well plates. When the cells in the 6-well plate reached confluence, they were transferred to 25 cm cell culture flasks for large volume expansion. The cells were then split 1:3 for further passaging, and at least one proportion at each passage was frozen in nitrogen for later use. The heterozygous FH KO in these cells at passages 10, 40 and 50 was verified by mutation analysis of specific PCR product sequencing.

### Chromosome preparation

When the cells reached 75% confluence, 0.5 μg/ml colchicine was added to the flask for 14 h, and the cells were harvested by 0.02% trypsinization. Then, the cells were centrifuged at 1500 rpm for 10 mins to obtain the cell pellet. Next, 6 ml of 0.075 M potassium chloride was added to the centrifuge tube, pipetted, and incubated at 37°C for 10 min. The cells were fixed with 1 ml of fixative (methanol/acetic acid=3:1) and centrifuged at 1500 rpm for 10 min. The supernatant was discarded, and 6 ml of fixative was added. The cells were mixed gently and then centrifuged at 1500 rpm for 10 mins. The supernatant was discarded again, and a few drops of fresh fixative were added, and the cells were mixed by pipetting. The fixative was removed before conventional G-banding after drying the slide at 75°C for 150 mins.

### Transmission electron microscopy

The cells were collected and fixed in 2.5% buffered glutaraldehyde (Servicebio, G1102). The cells were postfixed with 1% osmium tetroxide, dehydrated using a gradient series of ethanol and embedded in Epon 812 resin. The resin was polymerized for 48 h at 60°C. Ultrathin sections of 60–80 nm in thickness were prepared and poststained with 2% uranyl acetate and lead citrate. Images were acquired using a TEM (HT7700, Hitachi, Japan).

### Statistical analyses

All data represented at least three repeated experiments, and statistical analyses were performed using GraphPad prism 5.0. The results are shown as the means ± SD. Data were analysed by one-way ANOVA and Student’s t test (p< 0.05 was considered statistically significant), unless otherwise noted.

## References

[1] Anderson NM, Mucka P, Kern JG, Feng H (2018). The emerging role and targetability of the TCA cycle in cancer metabolism. Protein & cell.

[2] Chen L, Liu T, Zhou J, Wang Y, Wang X, Di W, Zhang S (2014). Citrate synthase expression affects tumor phenotype and drug resistance in human ovarian carcinoma. PloS one.

[3] Vander Heiden MG, Cantley LC, Thompson CB (2009). Understanding the Warburg effect: the metabolic requirements of cell proliferation. Science.

[4] Cascon A, Comino-Mendez I, Curras-Freixes M, de Cubas AA, Contreras L, Richter S, Peitzsch M, Mancikova V, Inglada-Perez L, Perez-Barrios A, Calatayud M, Azriel S, Villar-Vicente R (2015). Whole-exome sequencing identifies MDH2 as a new familial paraganglioma gene. Journal of the National Cancer Institute.

[5] Cardaci S, Ciriolo MR (2012). TCA Cycle Defects and Cancer: When Metabolism Tunes Redox State. International journal of cell biology.

[6] Morin A, Letouze E, Gimenez-Roqueplo AP, Favier J (2014). Oncometabolites-driven tumorigenesis: From genetics to targeted therapy. International journal of cancer.

[7] Tomlinson IP, Alam NA, Rowan AJ, Barclay E, Jaeger EE, Kelsell D, Leigh I, Gorman P, Lamlum H, Rahman S, Roylance RR, Olpin S, Bevan S (2002). Germline mutations in FH predispose to dominantly inherited uterine fibroids, skin leiomyomata and papillary renal cell cancer. Nature genetics.

[8] Li F, Zhang A, Shi Y, Ma Y, Du Y (2015). 1alpha, 25-Dihydroxyvitamin D3 prevents the differentiation of human lung fibroblasts via microRNA-27b targeting the vitamin D receptor. International journal of molecular medicine.

[9] Yu D, Zhong Y, Li X, Li Y, Li X, Cao J, Fan Z, Fan H, Yuan L, Xu B, Yuan Y, Zhang H, Ji Z (2016). Generation of TALEN-mediated FH knockout rat model. Oncotarget.

[10] Roy SG, Nozaki Y, Phan SH (2001). Regulation of alpha-smooth muscle actin gene expression in myofibroblast differentiation from rat lung fibroblasts. The international journal of biochemistry & cell biology.

[11] Goldsmith J, Levine B, Debnath J (2014). Autophagy, cancer metabolism. Methods in enzymology.

[12] Zhang Y, Fang N, You J, Zhou Q (2014). [Advances in the relationship between tumor cell metabolism and tumor metastasis]. Zhongguo fei ai za zhi = Chinese journal of lung cancer.

[13] Hirschey MD, DeBerardinis RJ, Diehl AME, Drew JE, Frezza C, Green MF, Jones LW, Ko YH, Le A, Lea MA, Locasale JW, Longo VD, Lyssiotis CA (2015). Dysregulated metabolism contributes to oncogenesis. Seminars in cancer biology.

[14] Pollard PJ, Wortham NC, Tomlinson IP (2003). The TCA cycle and tumorigenesis: the examples of fumarate hydratase and succinate dehydrogenase. Annals of medicine.

[15] Erez A, DeBerardinis RJ (2015). Metabolic dysregulation in monogenic disorders and cancer - finding method in madness. Nature reviews Cancer.

[16] Hainaut P, Plymoth A (2012). Cancer as a metabolic disease. Current opinion in oncology.

[17] Yan H, Parsons DW, Jin G, McLendon R, Rasheed BA, Yuan W, Kos I, Batinic-Haberle I, Jones S, Riggins GJ, Friedman H, Friedman A, Reardon D (2009). IDH1, IDH2 mutations in gliomas. The New England journal of medicine.

[18] Chen JQ, Russo J (2012). Dysregulation of glucose transport, glycolysis, TCA cycle and glutaminolysis by oncogenes and tumor suppressors in cancer cells. Biochimica et biophysica acta.

[19] Raimundo N, Baysal BE, Shadel GS (2011). Revisiting the TCA cycle: signaling to tumor formation. Trends in molecular medicine.

[20] Pollard PJ, Briere JJ, Alam NA, Barwell J, Barclay E, Wortham NC, Hunt T, Mitchell M, Olpin S, Moat SJ, Hargreaves IP, Heales SJ, Chung YL (2005). Accumulation of Krebs cycle intermediates and over-expression of HIF1alpha in tumours which result from germline FH and SDH mutations. Human molecular genetics.

[21] Aspuria PP, Lunt SY, Varemo L, Vergnes L, Gozo M, Beach JA, Salumbides B, Reue K, Wiedemeyer WR, Nielsen J, Karlan BY, Orsulic S (2014). Succinate dehydrogenase inhibition leads to epithelial-mesenchymal transition and reprogrammed carbon metabolism. Cancer & metabolism.

[22] Hoekstra AS, de Graaff MA, Briaire-de Bruijn IH, Ras C, Seifar RM, van Minderhout I, Cornelisse CJ, Hogendoorn PC, Breuning MH, Suijker J, Korpershoek E, Kunst HP, Frizzell N (2015). Inactivation of SDH, FH cause loss of 5hmC and increased H3K9me3 in paraganglioma/pheochromocytoma and smooth muscle tumors. Oncotarget.

[23] Janin M, Esteller M (2016). Oncometabolite Accumulation and Epithelial-to-Mesenchymal Transition: The Turn of Fumarate. Cell metabolism.

[24] Pfeiffer T, Schuster S, Bonhoeffer S (2001). Cooperation, competition in the evolution of ATP-producing pathways. Science.

[25] Yu M, Shi Y, Wei X, Yang Y, Zang F, Niu R (2009). Mitochondrial DNA depletion promotes impaired oxidative status and adaptive resistance to apoptosis in T47D breast cancer cells. European journal of cancer prevention : the official journal of the European Cancer Prevention Organisation.

[26] Sciacovelli M, Gaude E, Hilvo M, Frezza C (2014). The metabolic alterations of cancer cells. Methods in enzymology.

[27] Compton S, Kim C, Griner NB, Potluri P, Scheffler IE, Sen S, Jerry DJ, Schneider S, Yadava N (2011). Mitochondrial dysfunction impairs tumor suppressor p53 expression/function. The Journal of biological chemistry.

[28] Gaude E, Frezza C (2014). Defects in mitochondrial metabolism and cancer. Cancer & metabolism.

[29] Dong JT (2006). Prevalent mutations in prostate cancer. Journal of cellular biochemistry.

[30] Muller PA, Vousden KH (2013). p53 mutations in cancer. Nature cell biology.

[31] Brennan MS, Matos MF, Richter KE, Li B, Scannevin RH (2017). The NRF2 transcriptional target, OSGIN1, contributes to monomethyl fumarate-mediated cytoprotection in human astrocytes. Scientific reports.

[32] Carrasco-Garcia E, Moreno M, Moreno-Cugnon L, Matheu A (2017). Increased Arf/p53 activity in stem cells, aging and cancer. Aging cell.

[33] Jiang Y, Zhang M, Qian Y, Xu E, Zhang J, Chen X (2014). Rbm24, an RNA-binding protein and a target of p53, regulates p21 expression via mRNA stability. The Journal of biological chemistry.

[34] Lee SH, Kang YJ, Kim DH, Sung B, Kang JA, Chun P, Yoon JH, Moon HR, Kim HS, Chung HY, Kim ND (2014). A novel oxiranylchromenone derivative, MHY336, induces apoptosis and cell cycle arrest via a p53- and p21-dependent pathway in HCT116 human colon cancer cells. International journal of oncology.

[35] Chen Y, Pan K, Wang P, Cao Z, Wang W, Wang S, Hu N, Xue J, Li H, Jiang W, Li G, Zhang X (2016). HBP1-mediated Regulation of p21 Protein through the Mdm2/p53 and TCF4/EZH2 Pathways and Its Impact on Cell Senescence and Tumorigenesis. The Journal of biological chemistry.

[36] Wang Y, Blandino G, Givol D (1999). Induced p21waf expression in H1299 cell line promotes cell senescence and protects against cytotoxic effect of radiation and doxorubicin. Oncogene.

[37] Erhardt JA, Pittman RN (1998). Ectopic p21(WAF1) expression induces differentiation-specific cell cycle changes in PC12 cells characteristic of nerve growth factor treatment. The Journal of biological chemistry.

[38] Kotake Y, Naemura M, Murasaki C, Inoue Y, Okamoto H (2015). Transcriptional Regulation of the p16 Tumor Suppressor Gene. Anticancer research.

[39] Ben-Porath I, Weinberg RA (2005). The signals and pathways activating cellular senescence. The international journal of biochemistry & cell biology.

[40] Vaughan S, Jat PS (2011). Deciphering the role of nuclear factor-kappaB in cellular senescence. Aging.

[41] Braig M, Lee S, Loddenkemper C, Rudolph C, Peters AH, Schlegelberger B, Stein H, Dorken B, Jenuwein T, Schmitt CA (2005). Oncogene-induced senescence as an initial barrier in lymphoma development. Nature.

[42] Hornsby PJ (2007). Senescence as an anticancer mechanism. Journal of clinical oncology : official journal of the American Society of Clinical Oncology.

[43] Childs BG, Baker DJ, Kirkland JL, Campisi J, van Deursen JM (2014). Senescence and apoptosis: dueling or complementary cell fates?. EMBO reports.

[44] Debacq-Chainiaux F, Erusalimsky JD, Campisi J, Toussaint O (2009). Protocols to detect senescence-associated beta-galactosidase (SA-betagal) activity, a biomarker of senescent cells in culture and in vivo. Nature protocols.

[45] Kuilman T, Michaloglou C, Mooi WJ, Peeper DS (2010). The essence of senescence. Genes & development.

[46] Campisi J (2013). Aging, cellular senescence, and cancer. Annual review of physiology.

[47] Itahana K, Campisi J, Dimri GP (2004). Mechanisms of cellular senescence in human and mouse cells. Biogerontology.

[48] Baker DJ, Wijshake T, Tchkonia T, LeBrasseur NK, Childs BG, van de Sluis B, Kirkland JL, van Deursen JM (2011). Clearance of p16Ink4a-positive senescent cells delays ageing-associated disorders. Nature.

[49] Miwa S, Czapiewski R, Wan T, Bell A, Hill KN, von Zglinicki T, Saretzki G (2016). Decreased mTOR signalling reduces mitochondrial ROS in brain via accumulation of the telomerase protein TERT within mitochondria. Aging.

[50] Correia-Melo C, Marques FD, Anderson R, Hewitt G, Hewitt R, Cole J, Carroll BM, Miwa S, Birch J, Merz A, Rushton MD, Charles M, Jurk D (2016). Mitochondria are required for pro-ageing features of the senescent phenotype. The EMBO journal.

